# Functional Genomics Screening Utilizing Mutant Mouse Embryonic Stem Cells Identifies Novel Radiation-Response Genes

**DOI:** 10.1371/journal.pone.0120534

**Published:** 2015-04-08

**Authors:** Kimberly Loesch, Stacy Galaviz, Zaher Hamoui, Ryan Clanton, Gamal Akabani, Michael Deveau, Michael DeJesus, Thomas Ioerger, James C. Sacchettini, Deeann Wallis

**Affiliations:** 1 Department of Biochemistry and Biophysics, Texas A&M University, College Station, Texas, United States of America; 2 Department of Nuclear Engineering, Texas A&M University, College Station, Texas, United States of America; 3 Department of Veterinary Integrative Biosciences, College of Veterinary Medicine and Biomedical Sciences, Texas A&M University, College Station, Texas, United States of America; 4 Texas A&M Institute for Preclinical Studies, Texas A&M University, College Station, Texas, United States of America; 5 Department of Small Animal Clinical Sciences, Texas A&M University, College Station, Texas, United States of America; 6 Department of Computer Science and Engineering, Texas A&M University, College Station, Texas, United States of America; Rutgers University -New Jersey Medical School, UNITED STATES

## Abstract

Elucidating the genetic determinants of radiation response is crucial to optimizing and individualizing radiotherapy for cancer patients. In order to identify genes that are involved in enhanced sensitivity or resistance to radiation, a library of stable mutant murine embryonic stem cells (ESCs), each with a defined mutation, was screened for cell viability and gene expression in response to radiation exposure. We focused on a cancer-relevant subset of over 500 mutant ESC lines. We identified 13 genes; 7 genes that have been previously implicated in radiation response and 6 other genes that have never been implicated in radiation response. After screening, proteomic analysis showed enrichment for genes involved in cellular component disassembly (e.g. Dstn and Pex14) and regulation of growth (e.g. Adnp2, Epc1, and Ing4). Overall, the best targets with the highest potential for sensitizing cancer cells to radiation were *Dstn* and *Map2k6*, and the best targets for enhancing resistance to radiation were *Iqgap* and *Vcan*. Hence, we provide compelling evidence that screening mutant ESCs is a powerful approach to identify genes that alter radiation response. Ultimately, this knowledge can be used to define genetic variants or therapeutic targets that will enhance clinical therapy.

## Introduction

Radiation therapy is one of the primary treatment modalities for cancer. For example, up to 95% of breast cancer patients receive radiotherapy [1]. Enhanced efficacy could reduce side effects and improve outcomes. An understanding of the individual genes that make some people more sensitive or resistant to radiation could be used to tailor the dose or other therapeutics to the individual being treated. Several factors influence the efficacy of radiation therapy. In general, DNA repair systems and cell cycle checkpoints work together to maintain the genomic integrity of cells damaged by ionizing radiation. Much research has already been done on the molecular mechanisms that control the cellular response to radiation; however, the genetic basis of individual radiation response is not well understood. In fact, individual response to radiation is highly variable and can be considerably different depending on the dose range and rate [2]. There is evidence of linkage to specific chromosomal regions [3]; however, candidate gene studies have typically failed to discover variants associated with radiosensitivity. Further, while several genome wide association studies have been performed in humans and flies, the hits contribute only modest effect sizes [4].

A second factor complicating radiation therapy is the presence of radiation resistant cancer stem cells (CSCs). CSCs are a small, self-renewing sub-population of cells found in many cancer types. CSCs drive tumor development and metastasis, and most treatments including radiation therapy do not target CSCs. As it is these cells that are resistant to radiation, they contribute to treatment failure and relapse [5–8]. Therefore, specifically targeting CSCs would improve outcome for many patients.

Embryonic stem cells (ESCs) provide good models for CSCs. They share the same active transcription factors that regulate self-renewal and differentiation leading to tumor initiation and neoplastic proliferation [9–11]. One of the first studies to document this showed that genes associated with ESC identity were also expressed in poorly differentiated aggressive human tumors including breast, glioblastomas and bladder carcinomas [9]. Further works established the link between genes important for development and cancer by focusing on the diverse human epithelial cancers and c-Myc expression [12]. The well-defined pluripotency markers of ESCs such as Oct4, Sox2, Klf4, Nanog, and c-Myc have been shown to be commonly up-regulated in cancers and associated with tumor progression and increased risk of poor prognosis [13]. Further, like CSCs, ESCs are radiation resistant as they both employ the same mechanisms of self-protection including increased expression of DNA-repair pathways and resistance to reactive oxygen and nitrogen species [5,14].

Studying ESCs has several practical advantages over utilizing CSCs. ESCs are more readily isolated and have unlimited capacity for expansion, making them relatively easy to maintain in culture. In contrast, CSCs exist as a small population within a patient’s tumor, replicate infrequently, and are thus unable to be isolated and expanded to obtain the required numbers for study. ESCs have a stable genome and normal karyotype while cancer cell lines are constantly acquiring new genetic mutations. Also, ESCs are pluripotent and can be directed to differentiate into specific cell types *in vitro*. In contrast, the only known method for the full differentiation of CSCs without recurrence of carcinogenesis is their injection into an embryonic environment [15–18].

We utilized a novel screening approach in order to identify genetic factors involved in sensitivity and resistance to radiation in stem cells. As mutant ESC lines for virtually every protein-coding gene in the mouse genome are readily accessible [19–22], we selected and screened a subset of over 500 cancer candidate genes for radiation response. We screened for changes in both cell viability and gene expression. In order to be able to cross reference our two screening assays directly with one another on a gene-by-gene basis, we performed assays simultaneously for individual mutant clones 24 hours after irradiation. A similar screen for altered gene expression of gene traps in response to radiation identified 3 loci [23], but while it reports screening 6669 clones, there was no characterization of the diversity of their library, as they were not defined mutations from the outset. Hence, the fact that our library has pre-defined mutations is an asset. Two different radiation absorbed doses were screened; 0.5-Gy and 4-Gy. As these doses are not expected to cause cell lethality, especially in stem cells, we chose to evaluate viability and gene expression. We validated 13 clones as having altered phenotypes. Six of these genes have never been implicated in radiation response. Seven of the validated genes are already implicated in the literature as having a radiation response, thus confirming the validity of the screen. In particular *Klf8*, *Dstn* and *Map2k6* merit additional follow-up as down regulating these pathways enhances radiation response. Additionally, *Iqgap* and *Vcan* should be inhibited to enhance resistance or treat exposure in personnel exposed occupationally to radiation.

## Materials and Methods

### ES cell library and clone selection

Gene trap technology generates random loss-of-function mutations through insertional mutagenesis. High throughput gene trapping is a powerful technique that simultaneously integrates gene identification with expression and function into a single process [24]. We utilized ESC clones created by large-scale gene trapping in C57BL/6N mouse ESC [20]. Hansen et al. used high-throughput gene trapping with retroviral vectors in mouse C57BL/6N ESCs to generate a library containing 481,152 mutated ESC clones. The gene trapping construct contained a splice acceptor, a selectable marker gene (frequently a fusion of β-galactosidase and neomycin), and a polyadenylation signal that is placed within a retroviral genome such that it can be packaged into retroviral particles and used to infect cells. Using an automated, genomic-based inverse-PCR (IPCR) sequencing and annotation protocol they have produced a total of 532,829 insertion site sequence tags (ISTs) derived from 352,402 (or 73%) of these clones. When insertions occur within transcriptionally active regions, the marker gene is expressed and translated, allowing selection of mutant clones. Gene disruption is accomplished most often through the capture of endogenous gene transcription by the splice acceptor element within the trapping construct, or alternatively, by direct gene disruption as a result of insertion within an exon. Mutations have been identified in more than 10,000 unique genes and show a bias toward the first intron. The trapped ES cell lines, which can be requested from the Texas A&M Institute for Genomic Medicine, are readily available to the scientific community. Since many gene-trapping vectors contained β-geo, this allowed us to track the endogenous expression of the trapped gene through *β*-*gal*.

We focused our studies on a subset of ESC clones from our library and chose to evaluate cancer candidate genes utilizing the same gene list as The Cancer Genome Atlas (TCGA). As our gene trap library frequently contains multiple mutant clones for any given gene, all the clones were ranked based on relevant characteristics to identify the top clone for each gene in the library. Only clones that mapped to consistent genomic coordinates based on sequencing of PCR products adjacent to both ends of the insertion vector we considered. Then a Blast search was used to determine which matches were statistically significant (E-value < 10^-20^), and to rule out clones that mapped ambiguously to repetitive regions. Clones associated with a gene were required to disrupt at least 60% of the transcripts predicted for the gene, and to occur within the first half of the coding region (exons) of the gene of interest. Clones having these criteria were then ranked based on proximity of the insertion site to the start codon. We were able to identify 1614 unique clones in our library that both contained the β-*gal* marker inserted within the genomic region of the endogenous gene and were cancer candidate genes. To further narrow the list and enhance for novelty, we identified clones that represent genes that have been implicated in DNA repair, apoptosis, and/or cell cycle, and do not already have extensively developed mouse models based on data from the “Alleles and Phenotypes” tab in the Mouse Genome Informatics database (http://www.informatics.jax.org/). Since these genes were specifically selected to not have well-developed mouse models, they are relatively less known and studied than the more “classic” radiation response genes already identified in the literature. Ultimately, we identified 519 clones that were pulled from the library, thawed, expanded and assayed.

We utilized a number of different control clones for our assays on all plates within the course of our experiments. Wild type (WT) ESCs were used as a control for the cell death and viability assay. Changes in each clone’s viability due to radiation were normalized to the change in wild type clone viability at the same radiation dose. Since wild type ESCs do not contain a β*-gal* insert, they cannot be utilized for the evaluation of *β-gal* RLU; therefore, mutant ESC clones were used for controls in our gene expression assays. To assess positive response (increased gene expression) to radiation we utilized an ESC clone for *Bbc3* (*Puma*) a well-known radiation response gene [25]. Over the course of 22 separate experiments, we typically saw a mean 1.68 fold up-regulation in expression at 4-Gy (SEM = 0.12). *Sv2a*, a gene involved in synaptic vesicle fusion, was used as a negative control. Low-level expression of *Sv2a* was constant with or without irradiation.

### Cell culture

Initial clone recovery was asynchronous; however, it was clear that recovery, expansion, and synchronization was critical to simultaneous culture and subsequent assay performance. Uncoupling expansion and testing ultimately allowed for a more high-throughput process. Selected clones were thawed onto 24-well plates in ES media (DMEM, 15% FCS, 1× L-glutamate, 1×-Non-essential amino acids, 1×-nucleosides, 1×-Pen/Strep, 1×-2-Mercaptoethanol, 1mM sodium pyruvate, and 500U LIF). In order to maintain the undifferentiated state, clones were co-cultured with MEFs as a feeder layer, passaged every other day and maintained in media containing LIF. When cells were expanded and 85% confluent, they were frozen in quadruplicate. Upon re-thaw, these clones were more efficiently recovered and synchronized by 2–3 passages on MEFs followed by one passage on gel for subsequent use in assays.

### Radiation exposure

Two different radiation absorbed doses were selected with which to screen. We chose 0.5-Gy as a low clinically relevant absorbed dose, which is often also referred to as a hypersensitivity dose [24] and 4-Gy, a dose that is higher than is normally delivered in clinical treatments. Thawed cells were expanded and plated at forty thousand cells per well (96-well plate), cultured overnight and then exposed to 0 (control), 0.5, and 4-Gy of ionizing radiation from a Theratronics AECLT-780 Cobalt-60 Teletherapy unit at Texas A&M's College of Veterinary Medicine. A SSD manual technique was used for treatment planning utilizing a correction-based algorithm correcting back to calibration conditions. Briefly, the cell plates where set up 101 cm from the source and entirely contained within a 30 × 30 cm open field. The prescription point was set to target the dose to the top of the well. To ensure a homogeneous dose distribution across the entire area of the field, the cell plates where encased in tissue equivalent material (1.0 cm above and 0.5 cm below the plates) and the individual wells containing the cells within the plate were filled to maximum capacity with support media. In addition, the plates were positioned at least 2 cm from the edge of the field to minimize any charge particle disequilibrium created at the field edges. An ionization chamber positioned below the plate and an electrometer was used as a secondary dose verification to ensure the delivered dose was within intended tolerance levels. Twenty-four hours post-irradiation, cultures were harvested and assayed for differences in cell viability and gene expression.

### Cell death/viability assay

While clonogenicity assays remain the gold standard to evaluate radiosensitivity *in vitro*, they are not amenable to high throughput screens. Hence, cell death/viability assays have been used in screens. For example, Sudo et al., 2007 [26] utilized the sulforhodamine B (SRB) assay to evaluate cytotoxicity and survival fraction at 4 Gy after treatment of cells with RNAi in HEK293 cells. Zheng et al., 2008 [27] used ATP monitoring to assess the cytocidal, cytostatic and proliferative effects of 7.5 Gy radiation in glioblastoma cells after siRNA. Also, Choi et al., 2011 [28] used the MTT assay to validate radiosensitivity induced by loss of ELAV4. We utilized the Promega CytoTox-Glo Cytotoxicity Assay to determine cell death/viability after irradiation according to the manufacturer’s directions. This assay is a luminescent cytotoxicity assay that measures the relative number of dead cells in cell populations by measuring the extracellular activity of a distinct intracellular protease when the protease is released from membrane-compromised cells. The amount of luminescence directly correlates with the percentage of cells undergoing cytotoxic stress. Further, a relative number of dead and living cells can be calculated as well as a percent viability. The percent viability was calculated as the percentage of viable cells divided by the total number of cells as

$$\left(\text{\%viability = }\frac{\text{viable cells}}{\text{all cells}}=\frac{\text{all cells-dead cells}}{\text{all cells}}\right).$$

We used wild type ESCs as a control for this assay and all data were normalized to wild type.

### Gene expression assay

As the gene trap vector utilized to create our library contains *beta-gal*as a reporter gene,its activity can by assayed as a measure of endogenous gene expression of the trapped gene of interest. Reporter genes such as β-gal are routinely utilized to evaluate gene expression. The particular assay that we have employed is designed for rapid, sensitive, quantitative detection of β-gal in cell lysates. The wide dynamic range of the assay measures enzyme levels accurately from the femtogram to nanogram range. This assay has been previously validated, as it has been utilized to evaluate radiation responsive gene expression in gene-trapped ESCs [23]. We used the Applied Biosystem GalactoStar System according to the manufacturer’s directions to quantify the relative amount of *β-gal* expression per living cells by taking the relative light units of *β-gal* expression divided by the relative number of living cells as determined in the Cell Death/Viability assay above. Importantly, even trapped genes induced rapidly after irradiation would be expected to lead to increased β-gal activity at 24 h because β-gal is a stable protein.

### QC

After the initial round of testing we selected 18 genes/clones of interest. These clones were subjected to a rigorous QC analysis. Genomic sequencing was used to verify the exact insertion site of the vector within the gene of interest and Q-PCR verified the presence of one copy of the neomycin gene per genome to ensure single inserts. We also tested for presence of the *Sry* gene as a marker for the retention of the Y-chromosome in the cell line, and for contaminants such as mycoplasma and mold.

### Hit validation

We next wanted to validate radiation responsiveness in clones that passed QC by utilizing both time-course and dose-response studies for both gene expression and cell death/viability assays. For time-course experiments, cells were exposed to 4-Gy of radiation at 0, 6, 24, and 48 hours before being harvested for the assays. For dose-dependence experiments, cells were irradiated with doses of 0.5, 2, 4, 10 and 15-Gy and harvested 24 hours later.

### q-RT-PCR

RNA was extracted from cells using Qiagen RNeasy Mini kit, catalog number 74106 and quantitated by Nanodrop fluorimeter. We utilized QuantiTect SYBR Green RT-PCR Master Mix (Mat. number 1026225) to perform RT and PCR on the same reaction. QuantiTect Primer Assay gene specific primers for Gapdh, Vcan, Brca2, H3f3a, and Klf8 were used on a Stratagene Mx3005P System that ran the following cycling parameters: Reverse transcription for 30 min 50°C; PCR initial activation 15 min 95°C; followed by 40 cycles of Denaturation 15 sec 94°C, Annealing 30 sec 55°C, and extension 30 sec 72°C. Each RNA sample was amplified in triplicate and cycle thresholds (Ct) were determined automatically and delta Ct for each cell line were determined by subtracting the gene of interest Ct from the Gapdh Ct. Differences in delta Ct were determined between unirradiated and irradiated clones.

### RTCA analysis

Real Time Cell Analysis (RTCA) was performed using an ACEA Biosciences Inc., xCELLigence RTCA DP system on the six validated clones. The collected data detail the response of the mutant cells and wild type murine ESC for both 2-Gy and sham irradiations for a period of 120 hours post irradiation. The wells used for the mutant and controls were seeded with 10,000 cells each. Cell populations were measured as a function of time after irradiation to assess radiation effects on cell survival and cell index. Cell index was normalized at the time of radiation exposure.

## Results

### Identification of mutant clones for assays

We identified and selected 519 mutant clones that represent genes that have been implicated in DNA repair, apoptosis, and/or cell cycle. “Classic” radiation response genes, such as p53, were intentionally avoided so that we could identify novel genes. Clones were pulled from the library, thawed, expanded and assayed. Data on 386 mutant clones are presented in S1 Table for both assays at 0.5 and 4-Gy. As each assay was done in duplicate, data are given for the average of the duplicates for change in % viability and the average log ratio of *β-gal* activity normalized to the number of live cells determined by the Cyto-Tox Glo assay. Further, we calculated correlation scores between the duplicates for each experiment. The correlation score for the Cyto-Tox Glo assay is 0.72 and the *β-gal* assays is 0.98 indicating that our data are robust.

### Identification of genes associated with changes in viability after irradiation

We selected three genes (Brca2, Cdc25a, and Rpa1) to both validate the Cyto-Tox Glo assay and allow for cross-platform comparison. Based on reports in the literature, we demonstrate the anticipated phenotypes in our mutant ESCs (S1a Fig.). Depletion of BRCA2 by siRNA leads to cells that were able to overcome the normal G2 arrest after irradiation of U2OS cells at 6 Gy [29]. Hence, we see an increase in viability by up to 5% in ESCs. Cdc25a is well studied for its role in cell cycle check point regulation; it is required for progression from S to G2 phase [30]. When cells are exposed to radiation, Cdc25a is degraded and the cell cycle is arrested. Hence, loss of Cdc25a results in delay of cell-cycle progression such that cells can repair the damage. As a result, we observed increases in viability 24 hours after irradiation by 5%. We also observed decreased viability in ESCs with mutant Rpa1 by over 8%. Previous studies have shown that siRNA to Rpa1 results in increased radiosensitivity as measured by decreased survival fraction and halted cell cycle progression at G2/M phase [31]. Note that by comparison, the wild type clone has a loss of viability of less than 3% even at 4 Gy.

**Fig 1:**
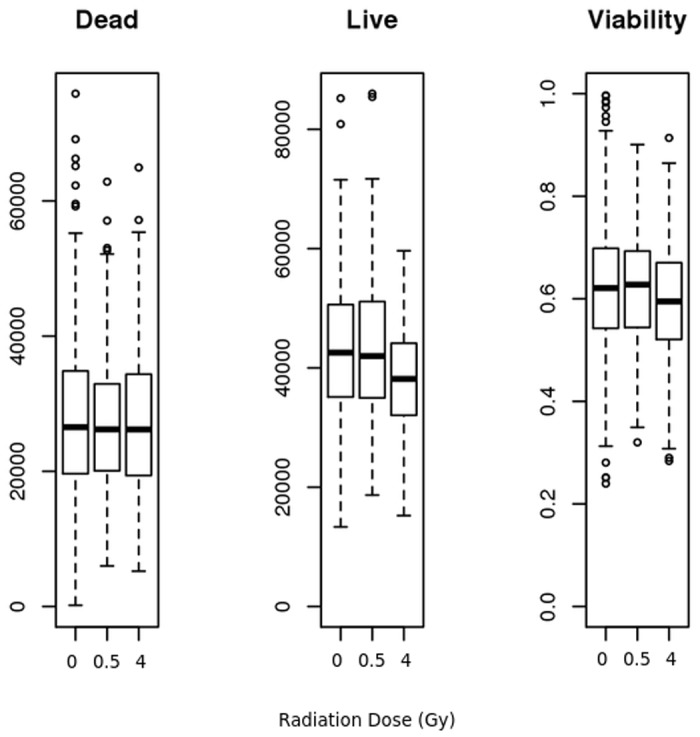
Cytotoxicity assay performance. Estimate of Dead cell relative light units (RLU), Live cell RLU, and % Viability for all clones at 0-Gy, 0.5-Gy and 4-Gy irradiation. While dead cell RLUs do not show statistically significant changes, changes in live cell RLUs and % Viability at 4-Gy are statistically significant indicating that as a whole, clones show fewer live cells and decreased viability at 4-Gy, presumably due to cell cycle arrest and decreased cell division.

During screening, we did not observe large differences in radiation induced cell death based on genotype, possibly due to the inherent radiation resistance of stem cells (Fig. 1). However, we did observe moderate changes in live cell numbers and % viability (Fig. 1) due to radiation (paired t-test of difference in % viability over all clones, 0-Gy vs 4-Gy, p < 0.00001). Such results can be explained by an arrest in cell growth induced by radiation. Mean viability (*μ*_wt_) for wild type cells was around 0.6 for all treatment conditions (0-Gy, 0.5-Gy, and 4-Gy). Changes in viability (Δ*V*) from 0.5 and 4-Gy to 0-Gy (i.e., Δ*V* = V_4Gy_—V_0Gy_) Δ *viability* = % *viability*_*4Gy*_—% *viability*_*0Gy*_), were calculated for all clones as well as for 22 wild-type replicate samples. To identify genes associated with changes in viability, Z-scores were calculated for all clones relative to the wild type as

$$z=\frac{\Delta V\text{-}\mu_{wt}}{\sigma_{wt}}$$

where μ = mean and σ = standard deviation. Thus, wild type samples represent the variability expected due to chance, and those clones that significantly deviate from this distribution are likely to be associated with a response to radiation. In fact, a histogram of the Z-scores shows a standard Normal distribution (black line in S1b Fig.). To assess significance, p-values were calculated based on a two-tailed distribution of Z-scores. Using a p-value cut-off p < 0.01, we identified a total of 28 unique genes associated with significant changes in viability: 5 associated with decreased viability at 0.5-Gy, 16 associated with increased viability at 0.5-Gy, and 7 associated with increased viability at 4-Gy (Table 1). Of the 5 clones that exhibited a decrease in viability at 0.5-Gy, two are associated with a response to oxidative stress: *Nuclear respiratory factor 1 (Nrf1) and Peroredoxin 3 (Prdx3*). Of those that exhibited an increase in viability at 0.5-Gy, several are involved in controlling the cell cycle: *Minichromosome maintenance complex component 2 (Mcm2)*, *CASP2 and RIPK1 domain containing adaptor with death domain (Cradd)* and *retinoblastoma binding protein 5 (Rbbp5*). At 4-Gy, no genes were statistically significantly associated with loss of viability, but among the 7 genes associated with increased viability, 2 are cell-cycle proteins such as *cell division cycle 25c* (*Cdc25c*) and *Ras-related protein Rab-8a (Rab8a)*.

**Table 1:** Genes associated with significant changes in viability.

		Gene	Change % Viability	Z-Score	p-value
**Decreased Viability Hits**	**0-Gy vs 0.5-Gy**	Prdx3	-30.89%	-4.41953	9.89E-06
		Bcap31	-30.51%	-4.36329	1.28E-05
		Bach1	-22.49%	-3.15661	0.001596
		Nrf1	-22.37%	-3.13909	0.001695
		Vamp12	-22.07%	-3.09443	0.001972
**Increased Viability Hits**	**0-Gy vs 0.5-Gy**	Mcm2	25.78%	4.100149	4.13E-05
		Cradd	24.46%	3.901591	9.56E-05
		Rbbp5	22.74%	3.643607	0.000269
		Lims1	22.58%	3.619399	0.000295
		Ctnna1	19.96%	3.224992	0.00126
		Arl6ip5	19.87%	3.212631	0.001315
		Dpagt1	19.55%	3.164071	0.001556
		Rfx2	19.28%	3.123158	0.001789
		Thrap3	18.98%	3.078622	0.00208
		Casc3	18.37%	2.98651	0.002822
		Ttl	18.36%	2.985033	0.002835
		Nrip1	17.72%	2.88807	0.003876
		Mll3	17.69%	2.883578	0.003932
		Psmb10	16.18%	2.65656	0.007894
		Topors	15.87%	2.611167	0.009023
		Exo1	15.68%	2.581592	0.009835
	**O-Gy vs 4-Gy**	Cdc25c	39.77%	5.313806	1.07E-07
		Rab8a	36.36%	4.887382	1.02E-06
		Bclaf1	29.79%	4.065455	4.79E-05
		Bcl7a	23.51%	3.280737	0.001035
		Kif22	19.35%	2.760707	0.005768
		Cux1	19.16%	2.736151	0.006216
		Scpep1	17.91%	2.58025	0.009873

### Identification of genes associated with changes in gene expression after irradiation

To quantify the relative amount of the trapped gene’s expression we utilized the *β-gal* construct incorporated into the trapping vector and determined the relative light units of *β-gal* expression per well and divided by the relative number of living cells as determined in the Cell Death/Viability assay. We then determined the ratio of RLU at 0.5-Gy to 0-Gy and 4-Gy to 0-Gy and compared each clones value to itself at the various radiation doses. For plotting in graphs, these ratios were then converted to log scale and Z-scores were calculated by estimating the mean and standard deviation with the clones themselves. We used the robust RANSAC algorithm to help remove the impact of outliers on parameter estimates [32]. Significant hits are defined as those with p<0.01 and are detailed in Table 2 for each radiation dose. In total we identified 20 genes with significantly altered expression; 13 with decreased expression and 7 with increased expression. Only 3 genes are identified as significantly altered in both radiation doses: *BRCA1/BRCA2-containing complex*, *subunit 3 (Brcc3)*, *Peroxisomal biogenesis factor 14 (Pex14)*, and *Destrin* (*Dstn*).

**Table 2:** Genes associated with significant changes in gene expression.

		Gene	Log Ratio *β-Gal*	Z-Score	p-value
**Decreased Expression Hits**	**0-Gy vs 0.5-Gy**	Brcc3	-1.77153	-12.4129	0
		Grb7	-0.62621	-4.24135	2.22E-05
		Nap1l4	-0.51406	-3.44121	0.000579
		Cep68	-0.51189	-3.42576	0.000613
		Anapc7	-0.50525	-3.37836	0.000729
		Rfx2	-0.45217	-2.99962	0.002703
		Rhbdd3	-0.40548	-2.66652	0.007664
				
	**O-Gy vs 4-Gy**	Brcc3	-1.85002	-9.34039	0
		Ing4	-0.92325	-4.48835	7.18E-06
		Epc1	-0.89975	-4.36531	1.27E-05
		Mtap4	-0.89935	-4.3632	1.28E-05
		Atp2b1	-0.63371	-2.97248	0.002954
		Adnp2	-0.60024	-2.79726	0.005154
		Diablo	-0.59001	-2.74367	0.006076
**Increased Expression Hits**	**O-Gy vs 4-Gy**	Serinc3	1.180251	6.524403	6.83E-11
		Pex14	0.739167	4.21514	2.5E-05
		Dstn	0.468382	2.797459	0.005151
				
	**0-Gy vs 0.5-Gy**	Dstn	0.441173	3.374142	0.00074
		Pex14	0.383902	2.965529	0.003022
		Ptbp2	0.368237	2.853763	0.00432
		Prdx3	0.357476	2.776985	0.005487
		Ccnb1	0.351157	2.731898	0.006297
		Tnfaip8	0.345919	2.69453	0.007049

### GO term analysis

Analysis of GO term enrichment among significant hits reveals similarity in gene functions. Hits for increased and decreased viability and increased and decreased expression were evaluated separately. Hits for 0.5-Gy and 4-Gy were pooled in each of the four categories. The comparison takes the hits as input, and compares them against the 386 genes in the screen as a background set to see if there is over-representation of any GO term. Enrichment was further refined by making sure that there were at least 3 genes in the starting population set for each GO term and 2 hit genes represented for each GO term. Table 3 details the enriched GO terms and associated genes. It is not surprising that genes necessary for “mitochondrial organization” and antioxidant response (*Nrf1* and *Prdx3*) might be required for cell survival after radiation stress. It also makes sense that genes involved in microtubule anchoring (*Pex14*) and actin depolymerization (*Dstn*) might be upregulated so the cell could arrest division to repair; or that genes involved in suppressing apoptosis (*Prdx3* and *Tumor necrosis factor*, *alpha-induced 8*, *Tnaip8*) might be upregulated. Further, genes that regulate growth are plausibly down regulated. However, there is no obvious link between genes that increased viability and involvement in Golgi vesicle transport, and links between down regulating genes involved in the plasma membrane or the ubiquitin ligase complex are also unclear.

**Table 3:** Evaluation of hits for GO enrichment.

Phenotype	GO number	GO term	Genes	Total in population	Q-value
Increased Viability	GO:0044431	Golgi apparatus part	Cux1, Rab8a	8	0.0101
Increased Viability	GO:0048193	Golgi vesicle transport	Cux1, Rab8a	8	0.0325
Decreased Viability	GO:0007005	mitochondrion organization	Nrf1 and Prdx3	6	0.0021
Increased Expression	GO:0022411	cellular component disassembly	Dstn, Pex14	18	0.0053
Increased Expression	GO:0009607	response to biotic stimulus	Prdx3, Tnfaip8	11	0.0084
Increased Expression	GO:0043154	negative regulation of cysteine-type endopeptidase activity involved in apoptotic process	Prdx3, Tnfaip8	5	0.0084
Decreased Expression	GO:0000123	histone acetyltransferase complex	Epc1, Ing4	4	0.0081
Decreased Expression	GO:0005887	integral component of plasma membrane	Atp2b1, Diablo	6	0.0184
Decreased Expression	GO:0040008	regulation of growth	Adnp2, Epc1, Ing4	22	0.02
Decreased Expression	GO:0000152	nuclear ubiquitin ligase complex	Anapc7, Brcc3	7	0.0208

### Validation—Clone selection and QC analysis

From the original 386 clones, we selected 18 for further validation. Details on selected clones are shown in Table 4, which lists gene symbols, names, functions, their scores in the first round of assays, and the reason they were selected for further screening. Clones were selected based on one of five criteria: increases in % viability (*IQ motif containing GTPase activating protein* (*Iqgap1*), *UDP-N-acetylglucosamine—dolichyl-phosphate N-acetylglucosaminephosphotransferase (Dpagt1)*, *BCL2-associated transcription factor 1 (Bclaf1)*, *Cell division cycle 25 c (Cdc25c)*, *and Kinesin family member 22 (Kif22*)); decreases in % viability (*BTB and CNC homology 1 (Bach1)*, *Dynein light chain roadblock-type 1 (Dynlrb1)*, *Mitogen-activated protein kinase kinase 6 (Map2k6)*, *Wings apart-like homolog (Wapal)*, and *heterogeneous nuclear ribonucleoprotein A3 (Hnrnpa3*)); gene up-regulation in response to radiation (*Versican* (*Vcan*) and *Destrin* (*Dstn*)); gene down-regulation in response to radiation (*Inhibitor of growth family*, *member 4 (Ing4)* and *RNA binding protein with multiple splicing (Rbpms*)); and simultaneous changes in both % viability and in gene expression (*G1 to S phase transition 1 (Gspt1)*, *cut-like homeobox 1 (Cux1)*, *Krueppel-like factor 8 (Klf8)*, and *ATPase*, *Ca++ transporting*, *plasma membrane 1* (*Atp2b1*)). Of these 18 clones, only *Bach1*, *Dpagt1*, *Cdc25*, *Bclaf1*, *Cux1*, *Ing4*, *Atp2b1*, and *Dstn* had p- values < 0.01 and represent the more extreme phenotypes. Ten clones were selected based on moderate phenotypes. Additionally, some of these genes are involved in similar processes. For example, several are related to the cytoskeleton; *Dstn* is an actin depolymerizing factor and both *Dynlrb* and *Kif22* are involved in microtubule based movement. Several others are involved in cell cycle regulation and division: *Cdc25c*, *Bach1*, and *Wapal*.

**Table 4:** Selected radiation response genes.

			0.5 Gy		4 Gy		
Symbol	Name	Function	Delta % Viability	B-Gal Fold Change	Delta % Viability	B-Gal Fold Change	Reason Selected
Iqgap1	IQ motif containing GTPase activating protein 1	IQGAP may regulate cell morphology; C-terminal domain of IQGAP inhibited the GTPase activity of cdc42	7.67	-1.48	-2.19	-1.58	Increased Viability
Dpagt1	Dolichol phosphate GlcNAc-1-phosphate transferase	GlcNAc-1-P transferase, target of Wnt/beta-catenin signaling pathway	19.55	-2.01	7.06	-1.78	Increased Viability
Bclaf1	BCL2-associated transcription factor 1	induction of apoptosis, negative regulation of transcription, DNA-dependent, a transcriptional repressor that interacts with BCL2-related proteins	6.93	-1.29	29.79	-1.70	Increased Viability
Cdc25c	cell division cycle 25C	cell cycle, cell division, phosphoprotein phosphatase activity	9.40	1.19	39.77	-1.39	Increased Viability
Kif22	kinesin family member 22	DNA repair, microtubule-based movement; may play a role in regulating the movement of chromosomes along microtubules during mitosis	14.46	-1.02	19.35	-1.60	Increased Viability
Bach1	BTB and CNC homology 1	negative regulation of transcription from RNA polymerase II promoter	-22.49	1.35	-21.38	1.34	Decreased Viability
Dynlrb1	dynein light chain roadblock-type 1	microtubule-based movement, transport	-15.96	-1.13	-20.32	1.57	Decreased Viability
Map2k6	mitogen-activated protein kinase kinase 6	Serine/threonine-protein kinase	3.51	1.69	-10.32	2.18	Decreased Viability
Wapal	wings apart-like homolog (Drosophila)	cell cycle, cell division; oncogene, malfunction of the WAPL pathway may activate an S phase checkpoint or other apoptotic pathway leading to cell death	5.47	-1.16	-12.55	1.13	Decreased viability
Hnrnpa3	heterogeneous nuclear ribonucleoprotein A3	mRNA transport	-15.45	1.25	-18.12	1.16	Decreased viability
Vcan	Versican	A proteoglycan, interferes with CD44/ErbB-dependant signaling; specifically interacts with hyaluronan	-2.98	1.21	-3.92	2.30	Increased Expression
Dstn	Destrin	actin depolymerizing factor	6.00	2.76	-6.00	2.94	Increased Expression
Ing4	Inhibitor of growth familiy member 4	tumor suppressor, apoptotic process, cell cycle; regulates brain tumour angiogenesis through transcriptional repression of NF-kappaB-responsive genes	-1.00	-1.10	-8.00	-8.38	Decreased Expression
Rbpms	RNA binding protein with multiple splicing	RNA-binding protein: marker for retinal ganglion cells; positive regulation of pathway-restricted SMAD protein phosphorylation	12.39	-1.52	-0.38	-2.31	Decreased Expression
Gspt1	G1 to S phase transition 1	involved in translation termination	-5.57	-1.48	9.61	-3.61	Increased Viability; Decreased Expression
Cux1	cut-like homeobox 1	intra-Golgi vesicle-mediated transport	2.12	1.08	19.16	-2.46	Increased Viability; Decreased Expression
Klf8	Kruppel-like factor 8	promotes tumor invasion	5.29	1.00	13.34	-3.08	Increased Viability; Decreased Expression
Atp2b1	ATPase, Ca++ transporting, plasma membrane 1	ATP binding, hydrolase activity, Calcium ion transport	13.90	-1.39	14.00	-4.30	Increased Viability; Decreased Expression

**Fig 2:**
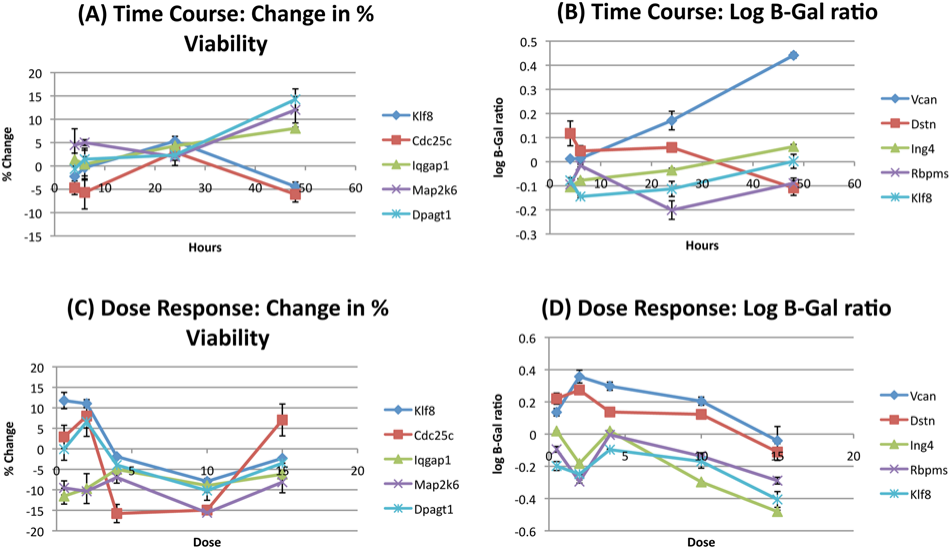
Validation assays. (A) Change in % viability over time for selected clones. (B) Change in gene expression over time for selected clones. (C) Change in % viability due to radiation dose for selected clones. (D) Change in gene expression due to radiation dose for selected clones. Error bars indicate SEM.

Clones of interest were subjected to QC assays where we verified the exact insertion site of the mutagenic gene trap and confirmed that they had a single insertion. Then they were progressed to further secondary assays. The subsequent data for each clone is summarized in S2 Table. For time course experiments, ESCs were exposed to 4-Gy irradiations at 4, 6, 24, and 48 hours before being harvested for the assays. The 48 hour time point shows the greatest range in change in % viability and extends from-6% to + 14% for *Cdc25c* and *Dpagt1* respectively (Fig. 2a). *Map2k6* and *Iqgap* also show increased viability (12.7 and 8.1% respectively) at 48 hours. In terms of gene expression, the 48 hour time point also has the most range primarily due to *Vcan* with a 2.76 fold increase (log (2.76) = 0.44) (Fig. 2b). Otherwise, the other time points do not provide much range in terms of dramatically altered gene expression.

For dose-dependence experiments, cells were irradiated with doses of 0, 0.5, 2, 4, 10 and 15-Gy and harvested 24 h later. As expected, when we assessed changes in % viability the most noticeable result is that as radiation doses increase, cell viability decreases (Fig. 2c). In fact, all clones saw a drop in % viability at 4 and 10-Gy, though at 15-Gy *Cdc25c* saw an increase in viability of 7%. We see the most range in changes in % viability at the lower doses of radiation (0.5-Gy and 2-Gy where we see changes +/- 11%). At low doses of radiation, both *Klf8* and *Cdc25c* clones show increases of 12 and 8% viability respectively. While this data seems contradictory with the above observation of *Cdc25c* having decreased viability, it is important to note that at 4-Gy, *Cdc25c* has decreased viability. Again, this is consistent with time course data.

Evaluation of gene expression indicates that at high levels of radiation, gene expression is reduced (Fig. 2d). *Ing4* and *Klf8* are reduced by as much as 3 and 2.5 fold at 15-Gy. At 4-Gy, gene expression is not changed by more than 2 fold for any of the clones. In general *Ing4*, *Rbpms*, and *Klf8* show decreased gene expression with radiation, while *Vcan* consistently shows increased gene expression except at the very high dose of 15-Gy. *Dstn* shows increases at low dose radiation and decreases at high dose radiation. Further, we see that 2-Gy provides the most dynamic range for changes in gene expression (from 2 fold decreases to 3 fold increases). These data stress that changes in gene expression are sensitive to the dose of radiation.

In general, our secondary assays were able to confirm 13 clones we had identified as having altered responses to radiation in the first round of assays did in fact test positive in a repeat test. These genes included: *Iqgap*, *Dpagt1*, *Klf8*, *Cdc25c*, *Bach1*, *Dynlrb1*, *Map2k6*, *Vcan*, *Dstn*, *Ing4*, *Rbpms*, *Wapal*, and *Hnrnpa3*. Five of these had been identified as having statistically significant changes (*Dpagt1*, *Cdc25c*, *Bach1*, *Dstn*, and *Ing4*).

We have further validated expression of some radiation response genes and a few of our hits by q-RT PCR in wild type (WT) ESCs after radiation. We chose to utilize WT ESCs to show that the trapping construct wasn’t introducing any artifacts into the mutant cells. We utilized Gapdh for normalization (calculation of dCt) as has been done by several other manuscripts that utilize q-RT PCR to detect changes in gene expression due to radiation [33,34]. We then compared unirradiated and irradiated samples (to calculate ddCt) to determine changes in gene expression. Again, we see the expected changes in expression. We evaluated Vcan by q-RT PCR and see increases in expression of over 4 fold at 4 Gy (S2 Fig.). Our previous data from the β-gal assay suggested changes of over 2 fold (Fig. 2d). The literature also confirms this finding as *Vcan* is consistently upregulated both in our studies and in the literature [35]. BRCA2 has been shown to decrease in expression after irradiation [36]. We see expression at 4 Gy drop to 0.73 fold the level observed without radiation by q-RT PCR (S2 Fig.). Data from the β-gal assay suggested decreased expression by almost 3 fold (data not shown). H3F3A has been shown to increase expression after radiation [37]. Accordingly, we see increases of up to 1.22 fold at 4 Gy by q-RT PCR (S2 Fig.) and1.5 fold by β-gal (data not shown). We also evaluated Klf8 expression by q-RT PCR and we see expression at 2 Gy drop to 0.77 fold the level observed without radiation by q-RT PCR. This was also observed by β-gal expression dropping approximately 2 fold (Fig. 2d). Hence, we see correlation in changes in gene expression between q-RT PCR and β-gal.

### RTCA analysis

**Fig 3:**
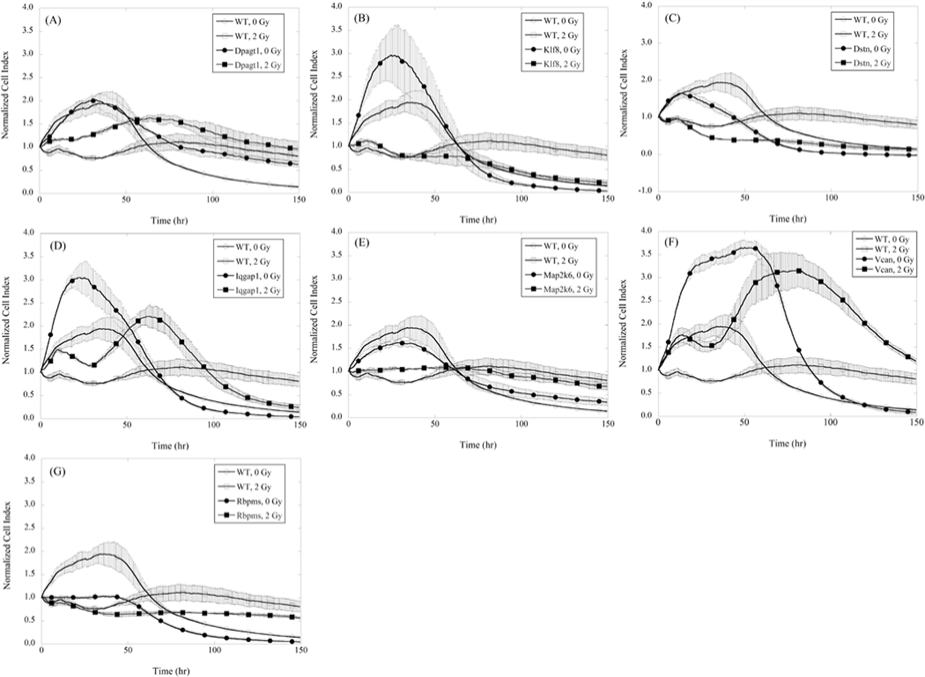
Cell growth as a function of time for wild type (WT) and (A) Dpagt1-mutant ESCs, (B) Klf8-mutant ESCs, (C) Dstn-mutant ESCs, (D) Iqgap1-mutant ESCs, (E) Map2k6-mutant ESCs, (F) Vcan-mutant ESCs, (G) Rbpms-mutant ESCs. Cells were irradiated with a dose of 2-Gy just before incubation using x-rays. Error bars indicate SEM.

Seven of the validated mutant clones were selected for further analysis. Cell growth studies were carried out using a Real Time Cell Analyzer (RTCA, ACEA Biosciences, Inc., California) to assess their radiation response as a function of time. The power inherent in this particular assay is it allows us to follow the cell index (CI) continuously over several days such that we are allowed a more complete overview than the previous time course assay where we only were able to obtain 4 discreet time points. The results are presented in Fig. 3 and show the cell growth as a function of time after irradiation at 2 and 0-Gy (control/sham) for a period of 120 hours. The data indicate that gene traps even without radiation exposure result in changes in both the maximum CI and the time in which it is achieved. For example, WT cells achieve a maximum CI of 2 within 40 hours and then declined to a CI of approximately 0.5 at 100 hours. *Map2k6* clones only reach a CI of 1.5 and take about 20 hours to achieve. However, *Vcan* reached a CI of 3.5 within 48 hours. Irradiated WT ESCs became arrested with no significant change in cell population (CI ~ 1) as a function of time. The irradiated mutants behave differently and such data can be utilized to predict how using a therapeutic drug to inhibit these genes would result in increased (or decreased) response to radiation therapy. For example, we have 2 targets that when knocked down show enhanced sensitivity to 2-Gy radiation (in comparison to WT). *Dstn* is the best target. *Dstn* has a lower CI than WT even w/o radiation. ES cells with this gene trap show CI that do not recover after irradiation and our other assays (time and dose response) support this finding as we saw a change in viability of-10% at 2-Gy in our dose response assays and-7% at 4-Gy after 24 hour (S2 Table). *Map2k6* also shows enhanced sensitivity to 2-Gy over time. The RTCA data is further supported by our dose response data showing a change of-10% viability at 2-Gy. Further, we have identified clones that show enhanced resistance to radiation. *Iqgap* and *Vcan* clones both have cell indexes higher than WT and when exposed to radiation, the clones initially show a slight decrease in CI, but then the CI rebounds to a level even higher than the unirradiated WT clones. This rebound occurs at a late time point, past 48 hours which was the last time point we checked in the secondary assays. *Vcan* clones show significant resistance to radiation as the CI persists over 2 at 120 hours.

## Discussion

### Performance of the Screen

In order to define genes that both increase and decrease radiation sensitivity, we evaluated the ability to use a library of gene-trapped murine ESCs for identification of gene function and demonstrated that we were able to identify reproducible phenotypes. Eighteen clones were selected, 10 with rather moderate phenotypes, for additional studies. Of these, 13 repeated the phenotype of interest, a good indication that initial hits are not false positives. Further, of these 13 clones we have validated, 7 have been described in the literature as being involved in radiation response, this further indicates that our screen performs well. Consistent with our findings, Cdc25c repression prior to low-dose radiation induced more distinct hyper-radiosensitivity and prevented the development of induced radioresistance [38]. Literature on Bach1 indicates that inhibition of the protein protects against UVA induced damage [32], but does not necessarily indicate if that occurs through cell cycle arrest and apparent decreases in % viability as we have observed. Also consistent with our data, literature indicates that gamma irradiation leads to the activation of *Map2k6*, a cell cycle regulator that alters % viability as we have observed [39]. *Dstn* is reported to have decreased expression upon UV exposure [40]. While our studies show an increase in expression at low radiation doses, higher doses show decreased expression. This apparent discrepancy could be due to the different types of radiation, the different cell lines utilized, the total absorbed dose of radiation, or the time point after which the expression was assayed. *Vcan* consistently is upregulated both in our studies and in the literature [35]. *Ing4* was selected due to changes in expression, while the literature indicates that over-expression of *Ing4* increases radiosensitivity [41]. Finally, *Rbpms* is reported to be down-regulated in radiation-damaged cells which we also observed [42].

In comparison to other screening platforms, the most similar platform involves evaluating siRNA libraries for their effect on radiation response. There have been several small studies evaluating the effects of several hundred siRNAs per study on radiation response. One study targeted the human kinome with siRNA and while they identified BRCA2 as a key regulator of G2 checkpoint maintenance, they also noted that several kinases with known checkpoint roles were not identified [29]. The authors suggest that this is a reflection of the lack of siRNA-knockdown efficacy. Another study targeted genes involved in DNA damage repair by siRNA and also determined that loss of BRCA2 resulted in increased DNA damage in SQ20B cells after irradiation with 4 Gy [34]. In comparison, we find that loss of Brca2 in ESCs results in increased cell viability and decreased Brca2 expression. A third study evaluated siRNAs for 200 genes and response to 4 Gy by assaying cell survival with SRB [26] and found loss of genes such as H3F3A and ZDHHC8 had decreased viability. We also tested ESCs with targeted H3f3a and Zdhhc8 mutations for changes in viability. While we see decreased viability for each clone, the observed changes of 1.6% and 3.2% viability are rather modest and may reflect the fact that the ESCs contain heterozygous mutations (data not shown). Hence, while siRNA screens may have false negatives due to inefficient knockdown; comparatively, mutant ESC screens may have a more stringent threshold for activity and may not identify targets where low levels of activity are sufficient for response. However, our data indicate that ESC screens do identify genes where heterozygous loss leads to a more robust phenotype.

It is important to note that evaluation of siRNA libraries precludes simultaneous evaluation of specific targets for radiation induced changes in gene expression as we have done in our screen. Alternative platforms for evaluation of gene expression changes include microarray and RNA-Seq analysis. Generally speaking, RNA Seq data are extremely accurate with a false positive rate of < 2% [43]. And q-RT PCR results agree more closely with Illumina sequencing results than with microarrays [44]. We were able to see changes in H3f3a expression based on β-gal and q-RT PCR; it was identified in the literature through microarray [37]. Hence, while it is possible to anticipate that different results could occur due to the inherent difference between evaluation of mRNA levels verses evaluation of the stable β-gal enzyme; β-gal expression in mutant ESCs provides a reasonable assay to evaluate a specific gene’s expression in a high throughput manner.

### Importance of time and dose

The findings in our own secondary assays strongly support the importance of both dose and time dependence. For example, *Cdc25c* shows dramatic changes in viability based on absorbed dose (Fig. 2c). The RTCA assays follow individual clones over time and show that for genes such as *Vcan* and *Iqgap*, the short-term arrest that might look promising, is followed by a rebound that indicates radiation resistance. Both of these factors are likely to have significant clinical relevance and indicate that any genes identified in such screens must be carefully studied and put into a more clinical context.

In terms of screening, it also appears that evaluation of radiation effects at 24 hours is sufficient to identify genes of interest. The time course assays revealed that generally speaking we see changes in gene expression within 6 hours and while this tends to peak by 48 hours, evaluation at 24 hours is sufficient. Dose response assays indicate that utilization of 2-Gy yields the largest range. Further, 2-Gy is a clinically relevant dose which spans the threshold between inducing the most damage without activating the maximum amount of repair processes. Dose response assays also indicate that doses above 10-Gy are rather ineffective if the main goal is only inducing chromosomal aberrations that cannot be repaired and ultimately lead to the death or senescence. This analysis is reinforced both by our data, with doses of radiation up to 15-Gy where we were unable to detect any significant increases in cell death, and by numerous other studies that found high dose irradiation was no more effective than low dose irradiations [45–48].

### New radiation response genes identified

Our data indicate that the following 13 genes are involved in radiation response: *Iqgap*, *Dpagt1*, *Klf8*, *Cdc25c*, *Bach1*, *Dynlrb1*, *Map2k6*, *Vcan*, *Dstn*, *Ing4*, *Rbpms*, *Wapal*, and *Hnrnpa3* (Table 3). Half of these (*Cdc25c*[38], *Bach1*[32], *Map2k6*[39], *Dstn*[40], *Vcan*[35], *Ing4*[41], and *Rbpms*[42]) have been previously implicated in radiation response while the others (*Iqgap*, *Dpagt1*, *Klf8*, *Dynlrb1*, *Wapal*, and *Hnrnpa3*) have never been implicated in radiation response. These new genes will require additional study.

Of particular interest is the transcriptional repressor *Klf8* [49]. Over-expression of *KLF8* has been associated with a number of different cancer types including: ovarian carcinoma, renal cell carcinoma, hepatocellular carcinoma, breast cancer, gliomas, and gastric cancer [50–57]. KLF8 induces epithelial-mesenchymal transition (EMT), a hallmark of cancer invasion and metastasis. Further, KLF8 has been implicated in DNA-repair and is a factor contributing to therapeutic resistance [58]. It is reasoned that KLF8 could play a role in altering genomic integrity through aberrant DNA repair function and therefore contributing to the aggressive progression of cancer. Interestingly, its homolog, KLF4 prevents ESC differentiation and is important for the maintenance of self-renewal and pluripotency. Experimental evidence suggests that it could be therapeutically beneficial to patients undergoing radiation therapy to also receive a therapy that targets KLF8 activity. This is especially true considering a paper by Wang that demonstrated KLF8’s ability to promote human breast cancer cell invasion and metastasis by transcriptional activation of MMP9 [59].

It is important to note that even though we have utilized two separate phenotypes for screening (cell viability and gene expression) this does not imply that we have identified all types of radiation response genes. For example, induction of DNA damage response more often does not rely on induction of protein expression, but rather on posttranslational modifications, including phosphorylation, ubiquitinylation, etc. Hence, unless such genes have a direct effect on cell viability, the expression based screen that we utilized would not be able to identify them.

Using mutant murine ESCs has at least three additional advantages as well. First, given the similarities to cancer stem cells, they provide excellent models for determining gene function in cancer stem cells. Unfortunately, it is unknown how the genes we have identified function specifically in cancer stem cells and what role if any they might play in the maintenance of stemness or differentiation. Evaluation of these genes’ expression and function in populations of cancer stem cells would provide valuable information as to their utility as therapeutic targets. Unfortunately, acquisition of such cell populations is extremely difficult and outside the realm of this particular study. Second, power lies within the fact that ESCs can be differentiated. Once a gene is identified; specific clones can be utilized for differentiation immediately and evaluated in multiple cell types *in vitro*. Alternatively, a library of clones can be differentiated simultaneously in parallel and each clone screened individually. Finally, these ES cells can be used to generate mutant (knock out) mice. Three of the genes we identified do not currently have mouse models: (*Dynlrb1*, *Rbpms*, and *Hnrnpa3*). Also, none of the mouse models that do exist have been evaluated for radiation response. It is important to note that some of these null mice are embryonic lethal; however, heterozygous mice could be evaluated for phenotypes in response to radiation.

## Supporting Information

S1 Fig a) Change in % viability due to radiation dose for selected clones. Error bars indicate SEM. b.) Histogram of Z-score values of Δviability at 4 Gy calculated for the clones, relative to wild type cells. A standard Normal distribution (black line) shows the expected distribution of Z-scores based on the wild type population. Clones at the extremes (tails) are those most likely to be associated with a change in viability due to irradiation.
(TIF)

S2 Fig Radiation induced change in expression of selected genes as measured by q-RT-PCR in WT ESCs. Error bars indicate SEM.
(TIF)

S1 Table (XLSX)

S2 Table Secondary assay data. (XLSX)
