# Supplementary figures and images for: Functional Genomics Screening Utilizing Mutant Mouse Embryonic Stem Cells Identifies Novel Radiation-Response Genes
Source: PLoS One. 2015 Apr 8;10(4):e0120534. doi: 10.1371/journal.pone.0120534 (PMC4390347; doi:10.1371/journal.pone.0120534)

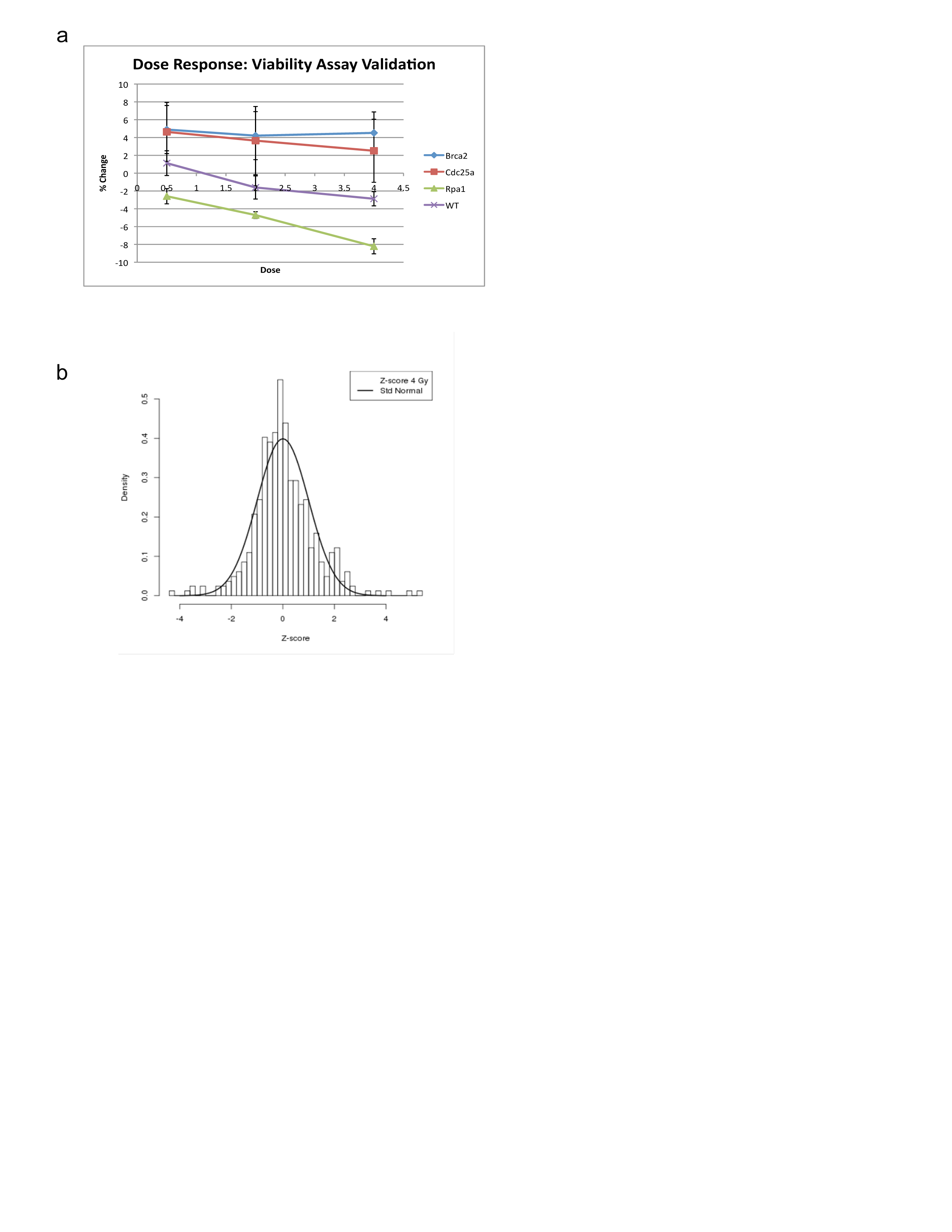

Supplement: S1 Fig — Error bars indicate SEM. b.) Histogram of Z-score values of Δviability at 4 Gy calculated for the clones, relative to wild type cells. A standard Normal distribution (black line) shows the expected distribution of Z-scores based on the wild type population. Clones at the extremes (tails) are those most likely to be associated with a change in viability due to irradiation. (TIF) [file pone.0120534.s001.tif]

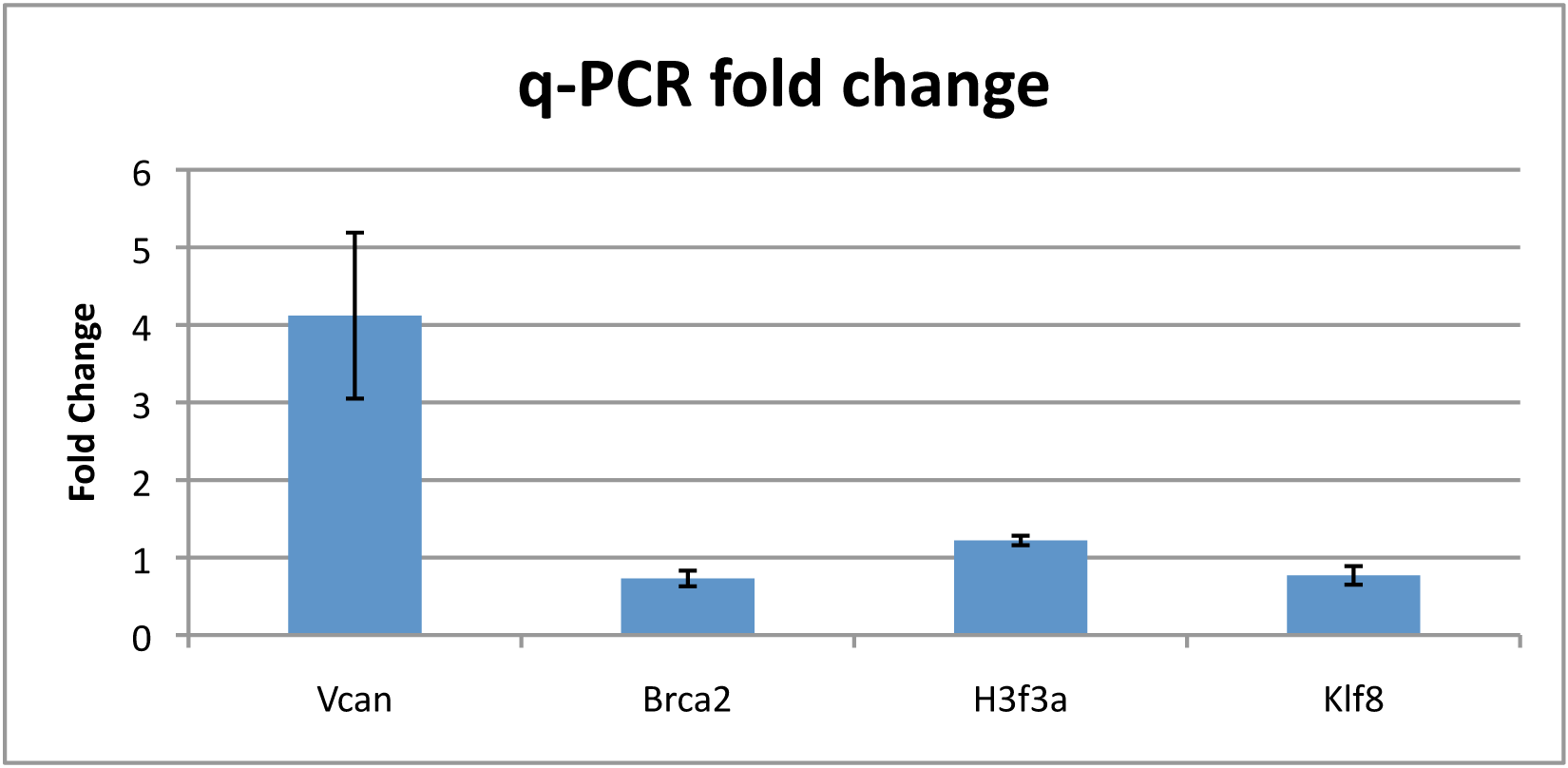

Supplement: S2 Fig — Error bars indicate SEM. (TIF) [file pone.0120534.s002.tif]
